# Overexpression of *CDSP32* (*GhTRX134*) Cotton Gene Enhances Drought, Salt, and Oxidative Stress Tolerance in *Arabidopsis*

**DOI:** 10.3390/plants9101388

**Published:** 2020-10-19

**Authors:** Mohammed Elasad, Adeel Ahmad, Hantao Wang, Liang Ma, Shuxun Yu, Hengling Wei

**Affiliations:** 1 Agricultural Research Corporation, Wad Medani P.O. Box 126, Sudan; asad_mo_saad@yahoo.com; 2 State Key Laboratory of Cotton Biology/Institute of Cotton Research, Chinese Academy of Agricultural Sciences, Anyang 455000, Henan, China; adeellqp@live.com (A.A.); wanghantao@caas.cn (H.W.); maliang@caas.cn (L.M.); yu@cricaas.com.cn (S.Y.)

**Keywords:** thioredoxins, abiotic stress, gene cloning, *Arabidopsis*, transformation

## Abstract

Upland cotton (*Gossypium hirsutum* L.) is the main natural fiber crop worldwide and is an essential source of seed oil and biofuel products. Many abiotic stresses, such as drought and salinity, constrain cotton production. Thioredoxins (TRXs) are a group of small ubiquitous proteins that are widely distributed among organisms. TRXs play a crucial role in regulating diverse functions during plant growth and development. In the present study, a novel *GhTRX134* gene was characterized and overexpressed in *Arabidopsis* and silenced in cotton under drought stress. Furthermore, the proline content and enzyme activity levels were measured in transgenic plants and wild-type (Wt) plants under drought and salt stress. The results revealed that the overexpression of *GhTRX134* enhanced abiotic stress tolerance. When *GhTRX134* was silenced, cotton plants become more sensitive to drought. Taken together, these findings confirmed that the overexpression of *GhTRX134* improved drought and salt tolerance in *Arabidopsis* plants. Therefore, the *GhTRX134* gene can be transformed into cotton plants to obtain transgenic lines for more functional details.

## 1. Introduction

Upland cotton (*Gossypium hirsutum* L.) is regarded as a vital natural fiber crop worldwide and is an essential source of seed oil and biofuel products [1]. Many abiotic stresses that negatively affect plant growth and development constrain cotton production, causing large economic losses [2]. Reports indicate that cotton production areas have decreased worldwide with continuing drought [1]. Therefore, drought-tolerant cotton varieties become the main objective for plant breeding programs [3]. Protein thiols are a part of proteins with sulfhydryl groups (–SH), which include thioredoxins (TRXs), proteins involved in glutathionylation, and glutaredoxins [4] play a critical role in biotic and abiotic stress tolerance [2,5]. TRXs are proteins that are widely distributed among organisms and can reduce the disulfide bridges of their target proteins by their active centers (cystein (Cys)-Gly-Pro-Cys) [6]. TRXs in plants are divided into six types (h, o, f, m, x, and y), according to their localization and function within the cell [7], four of which (f, m, x, and y) are chloroplastic proteins [8]. The chloroplastic drought-induced stress protein of 32 kD (*CDSP32*) is localized to the chloroplast [7]. *CDSP32* comprises two TRX domains (TRX-B) and belongs to a subfamily of TRXs that are specifically induced by environmental factors and oxidative stress [9]. TRXs are involved in diverse biological functions, such as enzymatic activation of Calvin cycle enzymes, regulation of protein activity, and abiotic stress responses [10]. A well-known example is light-induced activation of Calvin cycle enzymes such as fructose-1,6-bisphosphatase (FBPase) and NADP-malate dehydrogenase (NADP-MDH). TRXs are thioloxidoreductases with a pair of cysteine residues (Cys) that provide reducing power to diverse stress-related enzymes. Cys is regarded as a protective and regulatory mechanism of stress-related proteins [11], and it has been reported that diverse abiotic stresses increase free Cys levels [12]. Abiotic stress induces TRXs at the gene/or protein level. Nuruzzaman [13] reported that most TRX genes in rice were upregulated with drought and downregulated with cold stress. Proteomics research conducted on rice revealed that TRX genes were induced under copper stress [14]. NADPH-dependent thioredoxin reductase C (NTRC) is a protein that feeds electrons to TRXs; the overexpression of NTRC improved the tolerance of *Arabidopsis* to heat shock [15]. Several reports have confirmed that *CDSP32* is induced by abiotic and oxidative stress conditions [16]. TRXs have diverse mechanisms for protecting cells against oxidative stress, such as the upregulation of the expression of catalase and hydroperoxidase and the scavenging of radicals [17]. Several TRX targets are involved in the response to abiotic stress [18]. Additionally, TRXs have a role in regulating enzymes, which are involved in reactive oxygen species (ROS) scavenging processes such as catalase (CAT) and superoxide dismutase (SOD) [19]. Previously, we characterized the *GhTRX* gene family [2]. Among *GhTRX* genes, CotAD_54353 (*CDSP32*) (designated *GhTRX134*) belongs to the subfamily TRX-B, which contains two domains (TRX domain 1 and domain 2) [7]. Our results found that *GhTRX134* was highly induced under abiotic stress and phytohormone treatments, and thus, this gene was selected as an abiotic stress tolerance candidate gene [2]. To gain further insight into the functional role of *GhTRX134* (*CDSP32*), in this study *GhTRX134* was overexpressed in *Arabidopsis* and studied byvirus-induced gene silencing (VIGS) and yeast transformation. We found that the overexpression of *GhTRX134* enhanced drought, salt, and oxidative tolerance in *Arabidopsis*.

## 2. Materials and Methods

### 2.1. Plant Materials and Growth Conditions

Cotton (*Gossypium hirsutum* L. cv. CCRI10) CCRI10 was used for gene cloning and virus-induced gene silencing (VIGS). Seeds were germinated in a growth chamber room at 25 °C with a 16 h light/8 h dark cycle. *Arabidopsis* (ecotype Columbia-0) was selected for transgenic studies. Sterilized seeds were sown in ½-strengthmurashige and skoog medium (MS) and incubated at 4 °C for 3 days in dark conditions to break dormancy, and the plants were grown in a growth chamber at 22 °C, with a 16 h light/8 h dark photoperiod and a relative humidity of 80%.

### 2.2. qRT-PCR Assays

Total RNA was extracted with an RNAprep Pure kit (Tiangen, China). cDNA was synthesized using Prime Script RT Master Mix (TaKaRa, Japan). Quantitative RT-PCR was performed using SYBR Premix EX Taq Kit (TaKaRa, Japan) and an ABI 7500 Sequence Detection System (Applied Biosystems). The cotton and *Arabidopsis* actin genes were used as internal references [20]. The qPCR experiments were replicated at least three times, and the relative transcription levels were calculated using the comparative threshold cycle (CT) method [21].

The primers used for *GhTRX134* gene cloning, infusions, qPCR, and VIGS are shown in Supplementary Table S1. The primers used for qRT-PCR of the stress-responsive genes are listed in Supplementary Table S2. All primers were designed with OLIGO7 software and synthesized by GENEWIZ (Suzhou, China).

### 2.3. Gene Cloning and Sequence Analysis

The full-length coding sequence of *GhTRX134* was amplified using MightyAmp polymerase enzyme (TaKaRa, China). The PCR products were gel-purified and cloned into a simple pMD18-T vector (TaKaRa, Japan). The clones were transformed into *Escherichia coli*-competent cells and confirmed by sequencing (GENEWIZ, Suzhou, China). Total genomic DNA was isolated from cotton and *Arabidopsis* leaf tissues by the CTAB method [22]. The intron and exon structure was analyzed by comparing the genomic and coding sequences using expasy [2]. 

### 2.4. GhTRX134 Transgenic Arabidopsis Plants

#### 2.4.1. Construction of the *GhTRX134* Plasmids and Transformation of *Arabidopsis*

The full-length coding region of *GhTRX134* was amplified by PCR using forward and reverse primers with added Xba1 and Sac1 restriction sites. The *GhTRX134* DNA fragment was inserted into a pBI121 binary vector (Clontech, USA) digested with the same restriction enzymes. The pBI121 vector under the control of the 35S cauliflower mosaic virus (CaMV) promoter resulted in the 35S::*GhTRX134* construct. After being sequenced, the 35S::*GhTRX134* plasmid was transformed into *Agrobacterium tumefaciens* strain LBA4404 by the heat shock method [23]. Transgenic *Arabidopsis* plants were generated using the dip infiltration method [24]. The transgenic plants were selected using kanamycin (50 µg/mL) medium plates. Three out of seven T3 homozygous transgenic lines with the highest expression were chosen for further analysis.

#### 2.4.2. Growth of Transgenic Plants under Stress Treatments

T3 transgenic *Arabidopsis* plants and wild-type (Wt) seeds were grown on an *Arabidopsis* growth chamber for phenotypic comparisons and gene expression analysis. Various treatments (20% polyethylene glycol (PEG), 200 mM NaCl, cold, and 20 μM methyl viologen oxidative) were applied on two-week-old transgenic seedlings and Wt. Cold treatment was conducted in the dark by transferring the plants from normal conditions 22 °C to 4 °C for 72 h, whereas control plants were placed in the dark at 22 °C for 72 h. Samples: third–fourth rosette leaves were collected from both stress-treated and control plants after 0, 2, 6, 24, 48, and 72 h of drought, salt, oxidative, and cold stress treatments. Samples were frozen in liquid nitrogen, stored at −80 °C, and used for extraction of total RNA.

For phenotypic analysis, plant height, plant survival, and dry/wet ratio of transgenic and Wt plants were measured and calculated. Dry/wet ratio whole three-week-old seedlings grown under drought stress were separated and immediately weighed (fresh weight). The plants were kept at room temperature (on a petri dish) for 3 days and finally were completely dried for at least 12 h at 70 °C (dry weight). Dry/wet ratio for transgenic and Wt plants after drought treatment was calculated by dividing dry weight/fresh weight. Plant height (reduction %) in centimeters(cm) was measured from the soil surface to the top of both treated and untreated plants. Plant survival (%) was counted 20 days after methyl viologen(MV) treatment.

### 2.5. Plasmid Construction for VIGS Assay and Vector Inoculation

Specific primers containing the restriction sites Spe1 and Asc1 were used to amplify a443base pair (bp) fragment of *GhTRX134* via PCR and cloned into the pMD 18-T vector as described by the manufacturer (TaKaRa, China virus [25]). pMD 18-T was digested with the same restriction enzymes and then inserted into the pCLCrVA vector with the Spe1 and Asc1 restriction sites, as well as pCLCrVB, as a helper vector, and its derivatives pCLCrVA::*GhTRX134*, pCLCrVA::PDS, and pCLCrVA (control). All derivative plasmids were individually transferred into cotton cotyledons through *Agrobacterium* as described by Gu [26]. Phenotypes and gene expression were analyzed one month later. All steps were performed as described previously by Gao [25].

### 2.6. Analysis of Promoter Cis-Acting Elements 

The promoter region (1500 bp from the start codon) of *GhTRX134* was analyzed to detect the presence of cis-acting elements using the online search tool Plant CARE [2].

### 2.7. Trans-Activation Analysis of GhTRX134

The full-length open read frame (ORF), N-terminus, and C-terminus of *GhTRX134* were amplified using specific primers containing restriction (EcoR1and BamH1) sites and inserted into the pGBKT7 vector (digested by the same enzymes). The constructs pGBKT7-*GhTRX134*FL, pGBKT7-*GhTRX134*C, pGBKT7-*GhTRX134*N, and pGBKT7 (negative control) were transformed into the yeast strain AH109 following the Clontech method. The transformants were grown on synthetic dextrose medium lacking tryptophan (SD/-Trp) and synthetic dextrose medium lacking tryptophan, histidine, and adenine (SD/-Trp-His-Ade) medium, respectively.

### 2.8. Expression of Stress-Responsive Genes in Transgenic and Wt Plants under Abiotic Stress

The expression of five stress-responsive genes (*AtERD15*, *AtRAB18*, *AtRD22*, *AtRD29A*, and *AtKIN1*) was investigated in the transgenic L1, L2, L4, and Wt *Arabidopsis* plants under drought, oxidative, salt, and cold stress treatments.

### 2.9. Measurements of Proline Content, Enzyme Activity, and Chlorophyll Content

The fresh leaves of Wt and transgenic plants were harvested 5 days after drought and salt treatments. Proline content was detected using the sulfosalicylic acid method [27]. The enzymatic activities of CAT, SOD, and peroxidase (POD) were measured at 240 nm, 560 nm, and 470 nm, respectively, using a spectrophotometer according to [28]. Chlorophyll content was analyzed using 80% acetone, and the pigments a, b, and total chlorophyll were measured at 663 nm, 645 nm, and 652 nm, respectively, using a spectrophotometer as described by Lichtenthaler [29]. 

### 2.10. Statistical Analysis

All experiments were repeated three times with biological triplicates. Experimental data were presented in the form of the mean of three values with the standard deviation ± SD. The analysis for significance was performed using Student’s *t*-test (* *p* < 0.05, ** *p* < 0.01, and *** *p* < 0.001). Error bars are shown in all figures.

## 3. Results

### 3.1. Cloning and Characterization of GhTRX134 (CDSP32)

**Figure 1:**
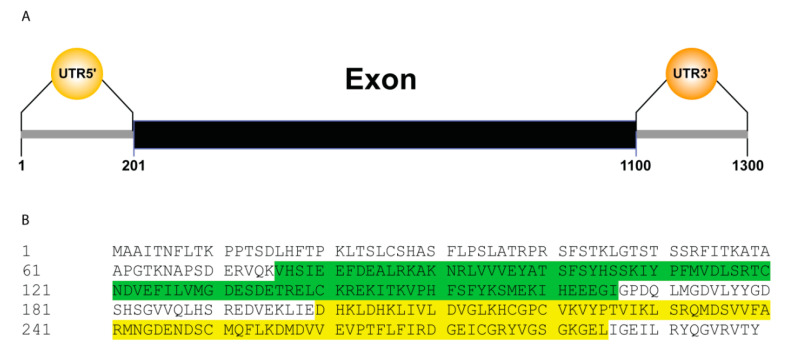
Gene structure was analyzed using expasy (**A**) Schematic structure of the genomic DNA showing exon (black block) and untranslated region (UTR) (grey lines) of *GhTRX134*. (**B**) The protein sequence with the two thioredoxins (TRX) domains (PF00085), 76-166 aa and 200-285 aa, are green and yellow highlighted, respectively.

The *GhTRX134* gene was cloned from a true leaf of *G. hirsutum*. According to genomic information, the *GhTRX134* gene contains one exon, and the ORF length is 900 bp and encodes a protein consisting of 299 amino acids, with a molecular weight of 32 kD. *GhTRX134* belongs to the subfamily TRX-B and contains two TRX domains (Figure 1).

### 3.2. Expression and Phenotypic Analysis of GhTRX134 Overexpression during Abiotic Stresses

To examine the specific function of *GhTRX134* in plants under drought, oxidative, salt, cold stresses, Wt, and three transgenic *Arabidopsis* lines (L1, L2, and L4) were selected for detection of the *GhTRX134* expression level and for phenotypic analysis. Generally, compared with the Wt plants, the transgenic plants under normal conditions showed slight growth retardation; this may be due to pleiotropic effects.

#### 3.2.1. *GhTRX134* Overexpression Enhances Drought Tolerance

**Figure 2:**
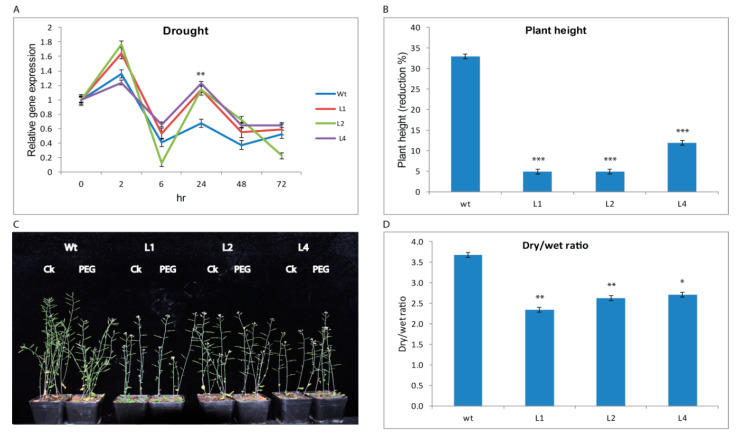
Overexpression of *GhTRX134* in *Arabidopsis* enhanced drought tolerance. Two-week-old plants were treated with 20% polyethylene glycol (PEG). (**A**) The expression of *GhTRX134* in transgenic lines and wild-type (Wt) under drought treatment was monitored by qRT-PCR. (**B**) Plant height was measured in centimeters (cm), and the percent reduction was calculated in transgenic lines and Wt plants under drought and control (CK) conditions. (**C**) Phenotypes of untreated (CK) transgenic and Wt plants compared with plants after drought treatment. (**D**) Differences in dry/wet ratios between transgenic plants and Wt plants after PEG6000 treatment; dry/wet ratio calculated by dry weight/wet weight of plants. The data represent the means of three replicates ±SDs. Asterisks indicate significance (* *p* < 0.05; ** *p* < 0.01; and *** *p* < 0.001) compared to Wt as determined by Student’s *t*-test.

Under drought stress (20% PEG6000), the expression of *GhTRX134* in transgenic lines was higher than that in the Wt lines at 2 and 24 h post-drought treatment (Figure 2A). Phenotypic analysis showed that, compared with the plants under the control conditions, the Wt plants had a high 6.8 centimeters (cm) reduction in plant height under drought stress. The transgenic L1, L2, and L4 plants showed a decreased reduction in plant height 0.6, 0.9, and 1.6 cm, respectively (Figure 2B,C). Additionally, the transgenic plants had a lower dry/wet ratio than the Wt plants (Figure 2D), and the high dry/wet ratio in Wt indicated relatively high water loss. Taken together, these results suggest that the overexpression of *GhTRX134* enhanced drought tolerance in *Arabidopsis*.

#### 3.2.2. *GhTRX134* Overexpression Confers Salt Tolerance

**Figure 3:**
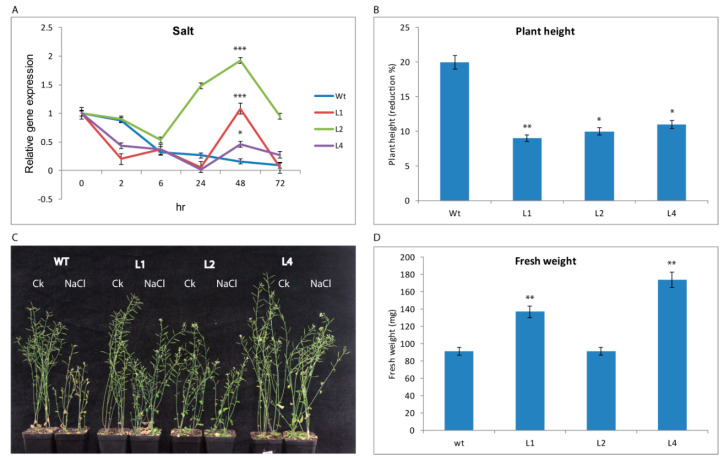
Overexpression of *GhTRX134* in *Arabidopsis* improved salt tolerance. Two-week-old plants were treated with 200 mM NaCl. (**A**) The expression of *GhTRX134* in transgenic lines and Wt under salt treatment was monitored byqRT-PCR. (**B**) Plant height was measured, and the percent reduction in transgenic lines and Wt plants was calculated under control and salt conditions. (**C**) Phenotypes of transgenic and Wt plants under control conditions (CK) compared with plants after salt treatment. (**D**) Fresh weight of transgenic lines and Wt plants after salt treatment. The data represent the mean of three replicates with ± SDs. Student’s *t*-test was used to detect significant differences (* *p* < 0.05; ** *p* < 0.01; and *** *p* < 0.001) compared to Wt.

For salt, *GhTRX134* was expressed more in the transgenic lines than the Wt plants at 48 h post-salt treatment (Figure 3A). Phenotypic analysis revealed that transgenic plants displayed a lower plant height reduction than the Wt plants, which exhibited a severe reduction in plant height under salt stress (Figure 3B,C). Additionally, transgenic plants had a higher fresh weight than the Wt plants (Figure 3D), suggesting that the overexpression of *GhTRX134* confers salt tolerance to *Arabidopsis*.

#### 3.2.3. *GhTRX134* Overexpression Improves Oxidative Resistance

**Figure 4:**
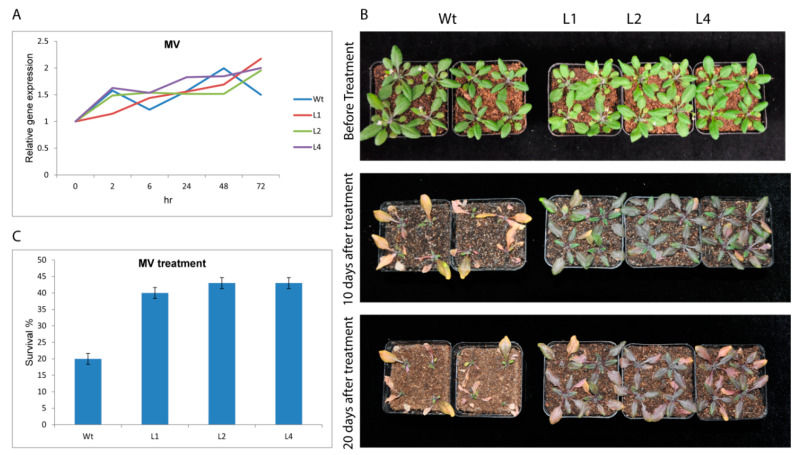
Oxidative stress analysis of transgenic lines and Wt plants (**A**) Expression level of *GhTRX134* in transgenic *Arabidopsis* and Wt was monitored by qRT-PCR. (**B**) Phenotypes of *GhTRX134*-overexpressing and Wt plants after treatment with 20 µM methyl viologen (MV) for 20 days. (**C**) The survival percentage of transgenic and Wt plants after oxidative stress. Values are the mean of three replicates ± SDs. Student’s *t*-test was used to determine the significant differences compared to Wt.

Under oxidative stress (methyl viologen(MV), USA), *GhTRX134* was induced in both transgenic and Wt plants but was slightly more induced in the transgenic plants than in the Wt plants at 72 h post-treatment (Figure 4A). Phenotypic analysis showed that the Wt lines showed more severe symptoms than the transgenic lines, which showed tolerance to MV (Figure 4B). In addition, compared with that of the Wt, which decreased, the survival percentage of the transgenic lines significantly increased (Figure 4C). These findings indicated that overexpression of *GhTRX134* confers oxidative stress resistance to *Arabidopsis*. 

For cold stress, transgenic lines and Wt plants were subjected to 4 °C for 72 h. The transcript level of *GhTRX134* was downregulated in transgenic plants but highly upregulated in Wt, with a peak at 24 h post-cold treatment (Supplementary Figure S1).

### 3.3. GhTRX134 Plays a Positive Role in Drought Stress Tolerence in Cotton Plants 

**Figure 5:**
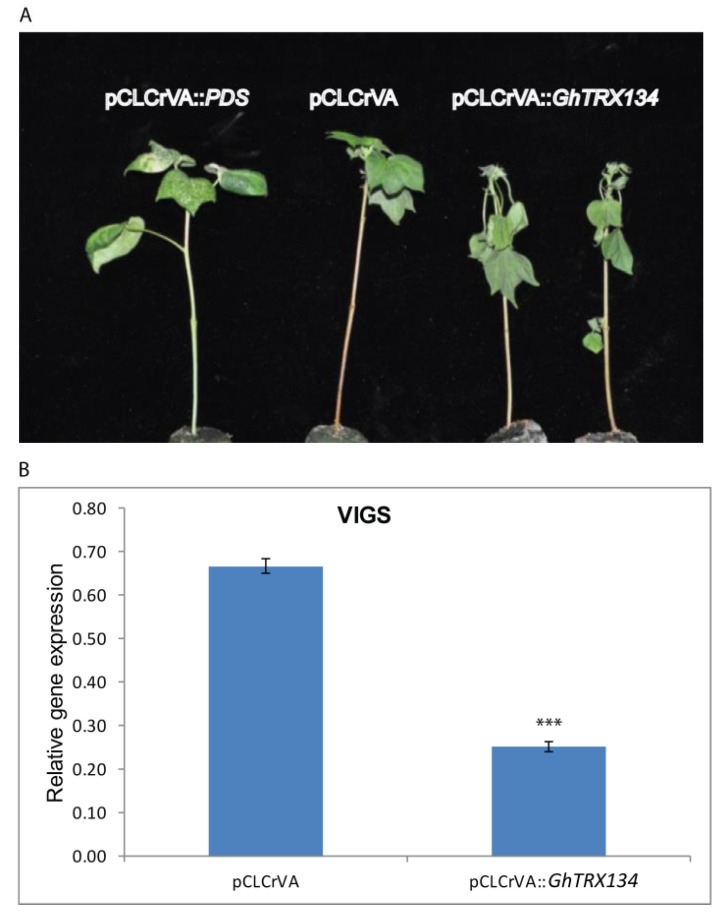
Silencing of *GhTRX134* using the pCLCrVA-pCLCrVB system inhibited tolerance to drought in cotton plants. (**A**) The phenotypes of the virus-infected cotton seedlings pCLCrVA::*GhTRX134*, the control pCLCrVA plants, and the pCLCrVA::PDS indicator plants. (**B**) The expression of *GhTRX134* in the virus-infected plants and the control (pCLCrVA) plants was examined by qRT-PCR. The values represent the means of three replicates ± SD. Student’s *t*-test was used to determine the significant differences (*** *p* < 0.001) compared to the control plants.

The VIGS (pCLCrVA-pCLCrVB) system was used to analyze the function of *GhTRX134* in cotton plants under drought stress in cotton plants injected with vectors harboring *GhTRX134*, infected plants harboring pCLCrVA::*GhTRX134*, indicator plants harboring pCLCrVA::PDS, and control plants harboring pCLCrVA. Phenotypic analysis for plants subjected to drought showed that the leaves in the infected plants displayed severe wilting, while the leaves in the control plants remained healthy (Figure 5A). The expression level of *GhTRX134* was detected by qRT-PCR when the leaves of the indicator (PDS) plants exhibited an albino phenotype. The expression level of *GhTRX134* in the infected plants (pCLCrVA::*GhTRX134*) was lower than that in the control plants (pCLCrVA) (Figure 5B). These results indicate a positive role for *GhTRX134* in drought tolerance.

### 3.4. In Silico Promoter Analysis

In silico analysis of the *GhTRX134* promoter we used, 1500 bp upstream sequences, were subjected to Plant CARE. The results revealed the presence of a protein-binding site (homeodomain leucine zipper, HD-Zip3) and sixMYB1-binding sites. In addition, cis-acting regulatory elements involved in circadian control (circadian) were detected (Supplementary Table S3). This result suggests that *GhTRX134* interacts with several transcription factors.

### 3.5. The GhTRX134 Gene Acts as an Activator

**Figure 6:**
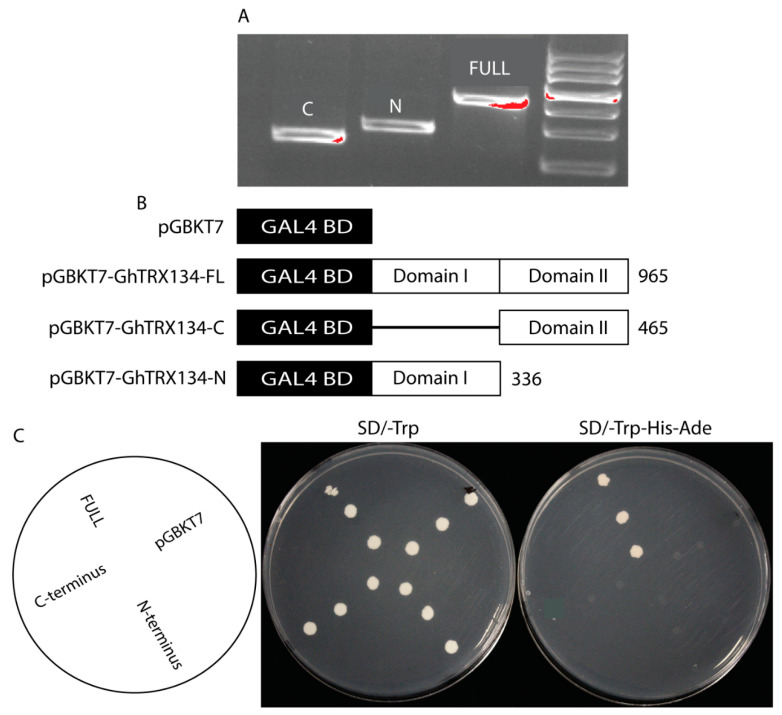
*GhTRX134* shows transcriptional activity. (**A**) The size of the *GhTRX134* fragments. (**B**) The yeastAH109 strain containing the pGBKT7 empty vector, pGBKT7-*GhTRX134*FL (full-length *GhTRX134*), pGBKT7-*GhTRX134*-N (N-terminal region of *GhTRX134*), and pGBKT7-*GhTRX134*-C (C-terminal region of *GhTRX134*). (**C**) Transformants were streaked on synthetic dextrose medium lacking tryptophan (SD-Trp) medium (control) and synthetic dextrose medium lacking tryptophan, histidine, and adenine (SD-Trp-His-Ade) medium (selective) to examine HIS3 and ADE2reporter gene activation. The media with yeast cells were incubated for 3 days at 28 °C.

To examine the transcriptional activity of *GhTRX134*, the pGBKT7 empty vector and the constructs of the *GhTRX134* gene were transformed into the yeast strain AH109. All transformants were grown on SD/-Trp. Yeast cells containing the pGBKT7 empty vector, pGBKT7-*GhTRX134*N, and pGBKT7-*GhTRX134*C did not grow on the selective medium (SD/-Trp-Ade-His). However, cells containing pGBKT7-*GhTRX134*FL grew on SD/-Trp-His-Ade (Figure 6), indicating that full-length *GhTRX134* functions as a transcriptional activator

### 3.6. GhTRX134 Overexpression Regulates the Expression of Stress-Responsive Genes

**Figure 7:**
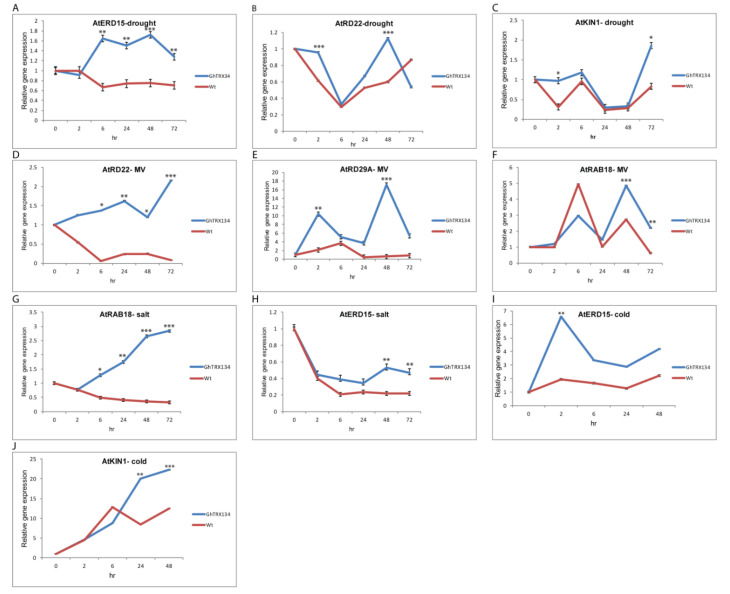
Expression of the *AtERD15*, *AtRAB18*, *AtRD22*, *AtRD29A*, and *AtKIN1* responsive genes in two-week-old transgenic and Wt*Arabidopsis* plants under drought, MV, salt, and cold. (**A**–**C**) Drought 20% PEG. (**D**–**F**) MV 20 µM. (**G**,**H**) Salt 200 mM NaCl. (**I**,**J**) Cold 4 °C for 72 h stresses was monitored by qRT-PCR. The *Arabidopsis thaliana* actin gene was used as a housekeeping gene. The comparative threshold cycle (CT) method was used to evaluate the relative expression; each value is the average of three replicates ± SDs. Student’s *t*-test was used to determine the significant differences (* *p* < 0.05; ** *p* < 0.01; and *** *p* < 0.001) compared to Wt.

To explore the molecular mechanisms of *GhTRX134* in abiotic stress, the expression of five responsive genes, *AtERD15*, *AtRAB18*, *AtRD22*, *AtRD29A*, and *AtKIN1*, was investigated in Wt and transgenic L1, L2, and L4 of *Arabidopsis*. The qRT-PCR analysis revealed that the expression levels of these genes were significantly enhanced in the transgenic plants under abiotic stress compared to the Wt plants. Under drought conditions, the genes *AtERD15*, *AtRD22*, and *AtKIN1* were more highly induced in the transgenic plants than in the Wt plants at 48 h, 48 h, and 72 h post-drought treatment, respectively (Figure 7A–C). For MV treatment, the expression of *AtRD22*, *AtRD29A*, and *AtRAB18* was upregulated in transgenic plants while downregulated in the Wt plants (Figure 7D–F). Under salt stress, the genes *AtRAB18* and *AtERD15* were highly induced in the transgenic plants compared to the Wt plants at 48 h post-salt treatment (Figure 7G,H). Under cold stress, the expression of *AtERD15* and *AtKIN1* was higher at 2 and 24 h in the transgenic plants than in the Wt plants (Figure 7I,J). Taken together, these results confirmed that the overexpression of *GhTRX134* improved the transcriptional levels of the stress-responsive genes

### 3.7. Measurements of Proline Content, Enzyme Activity, and Chlorophyll Content

**Figure 8:**
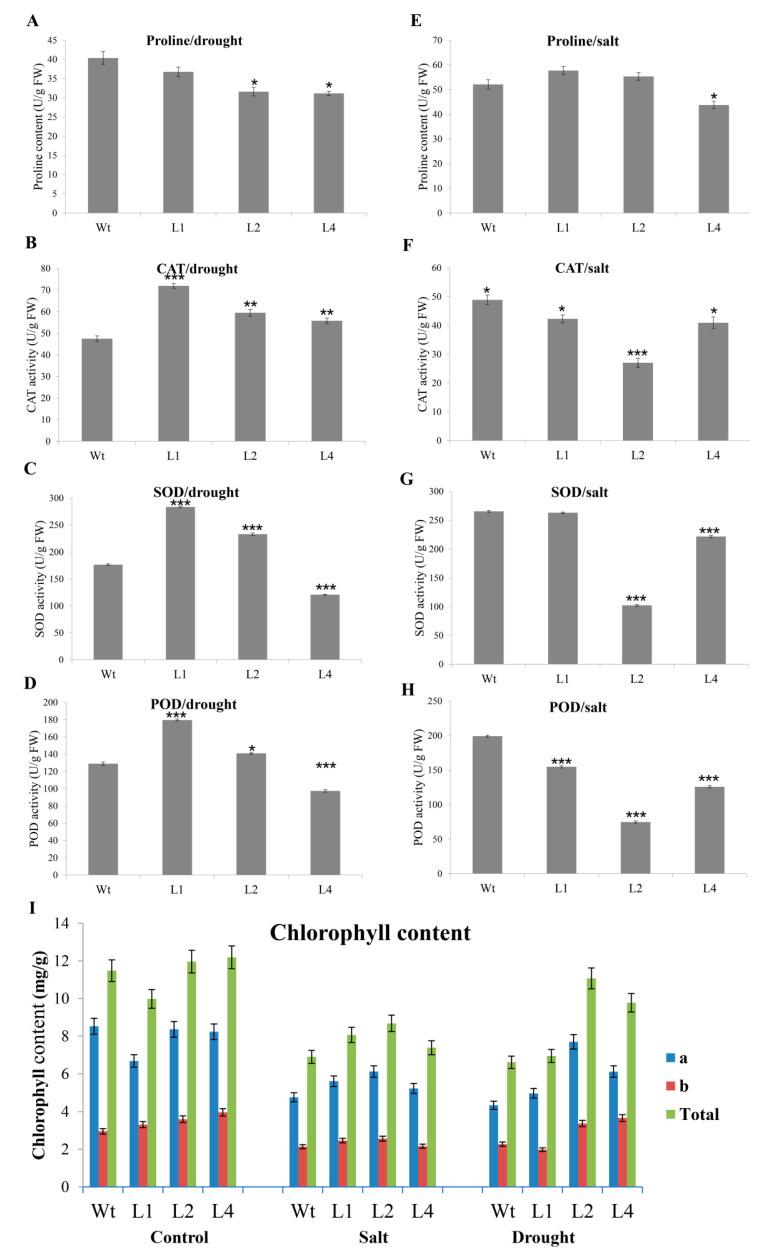
*GhTRX134* overexpression increases enzyme activities under drought conditions and proline content under salt stress. Two-week-old plants were treated with (**A**–**D**) 20% polyethyleneglycol to measurethe proline content, catalase (CAT), superoxide dismutase (SOD), and peroxidase (POD) enzyme activity levels. (**E**–**H**) 200 mM NaCl to measure proline content, CAT, SOD, and POD enzyme activity levels. (**I**) Chlorophyll content under salt (200 mM NaCl) and drought (PEG 20%). Proline and chlorophyll content were measured after drought and salt for 5 days using aspectrophotometer. The values represent the means of three replicates ± SDs. Student’s *t*-test was used to determine significant differences (* *p* < 0.05; ** *p* < 0.01; and *** *p* < 0.001) compared to the control plants.

The proline content and enzyme activities were investigated in Wt and transgenic *Arabidopsis* plants subjected to drought and salt stress. Under drought conditions, the transgenic plants showed reduced proline content (Figure 8A), while the activities of the CAT, SOD, and POD enzymes were induced (Figure 8B–D). In contrast, under salt stress, the proline content was slightly increased (Figure 8E), while the enzyme activities were reduced with respect to the Wt plants (Figure 8F–H). There were no significant differences in chlorophyll content between the transgenic lines and Wt plants under the control conditions. In contrast, the transgenic plants under salt and drought stresses showed higher chlorophyll content than the Wt plants (Figure 8I). These findings revealed that overexpression of *GhTRX134* significantly enhanced drought and salt tolerance in *Arabidopsis* via increased enzyme activities and proline content, respectively

## 4. Discussion

Plant growth and development are affected by environmental stresses. Therefore, to overcome these stresses, plants have developed various biochemical and physiological strategies [30]. The chloroplastic drought-induced stress protein of 32 kDa (CDSP32) is a TRX that is mainly involved in plastidic responses under oxidative stress [9]. *CDSP32* targets play a fundamental role in the protection against abiotic and oxidative stresses. The plastidic BAS1is considered the main target of *CDSP32*, which detoxifies peroxides [9]. *CDSP32* is a key factor in the tolerance to abiotic stress by serving as an electron donor to its targets [31]. In this study, we overexpressed *GhTRX134* in *Arabidopsis* to examine its function at the phenotypic, physiological, molecular, and chemical levels in transgenic plants under abiotic stress. The transgenic plants showed slight growth retardation compared to the Wt plants under normal growth conditions, which may be due to pleiotropic effects and agrees with previous reports [32,33]. In this work, the overexpression of *GhTRX134* improved drought tolerance in *Arabidopsis*, while silencing *GhTRX134* in cotton plants greatly reduced drought tolerance in cotton. The main physiological effect of drought stress on plants is the reduction in vegetative growth [4]. *GhTRX134* was highly induced under drought and the transgenic lines exhibited less plant height reduction and lower dry/wet ratio compared to Wt plants, indicating improved drought tolerance. This result is in agreement with the result from a previous report which revealed that *CDSP32* expression was highly induced in *Solanum tuberosum* plants tolerant to drought stress [16]. These results demonstrate the potential role of *GhTRX134* in drought stress. Indeed, *CDSP32* is induced during environmental stresses that result in a reduction in glutathione [16]. Therefore, *CDSP32* could constitute an electron donor to its target proteins to maintain the activity levels required for protection under abiotic stress [34]. Salinity is a key environmental factor that affects plant growth and causes a substantial loss in plant production worldwide [35]. Salinity is toxic to cell metabolism and has a harmful effect on the functioning of several enzymes. High salinity causes membrane disorganization, osmotic imbalance, inhibition of cell division and expansion, and reduction in growth [5]. In this work, the transgenic plants displayed less plant height reduction and higher fresh weights than the Wt plants, suggesting an involvement of this gene in salt tolerance. This result is in agreement with a previous study showing that *AtTRX*m2 was induced under NaCl treatment and enhanced *Arabidopsis* salt tolerance [36]. Oxidative stress is regarded as the main cause of cellular damage and cell death [37]. MV(paraquat) is aherbicide that is used to induce oxidative stress [38]. More than 56 plant-specific protein kinases were reported to be regulated by MV [39]. TRXs have several mechanisms for enhancing the cell abilities against oxidative processes, such as oxidized and reduced forms [17]. In this study, *GhTRX134* was induced by oxidative stress and the transgenic plants exhibited less severe oxidative stress symptoms than the Wt plants. This finding is consistent with the previous report showing that, in potato plants under oxidative stress, CDSP32 was highly induced, and the leaves exhibited slightly fewer bleached spots than the Wt leaves [9]. In addition, the induction of the *CDSP32* gene during oxidative stress accompanied tolerance to oxidative stress, while plants lacking *CDSP32* had increased sensitivity to oxidative stress [16]. Takemoto [17] revealed that *E. coli* TRX mutants become highly tolerant to oxidative stress, whereas TRX-deficient mutants displayed higher sensitivity than the Wt strain. Additionally, in yeast, the deletion of two TRX genes leads to pleiotropic effects, such as oxidative hypersensitivity [40].

The promoter is the upstream part of the gene that contains several cis-acting elements, which are specific binding sites for proteins involved in the regulation of transcription and initiation [6]. In this work, it was determined that the promoter of *GhTRX134* contains HD-Zip protein-binding sites, MYB1-binding sites, and cis-acting regulatory elements (circadian). Homeodomain leucine zipper (HD-Zip) proteins are transcription factors that contribute to diverse activities such as stimuli responses. HD-Zip functions depend on the activity of their target genes [41]. MYBs regulate their target genes via interaction with their promoters [42]. The circadian elements enhance responses to abiotic stresses such as drought and salt [43]. Our findings indicate that these transcription factors might be interacting with GhTRX134. This idea is consistent with the transcriptional activation assay, which indicated that full-length *GhTRX134* has transcriptional activation and possibly activates other genes.

Stress-responsive genes have been confirmed to be induced by abiotic stress, and their proteins participate in protecting cells from these stresses [32]. TRXs are involved in the regulation of the expression of numerous stress-regulated genes [44]. In this study, transgenic plants induced stress-responsive genes under various abiotic stresses. This result agrees with a previous result demonstrating that abiotic tolerance in transgenic *Arabidopsis* plants was improved by the increase in responsive gene products under stress conditions [45]. The products of numerous responsive genes counteract abiotic stresses by regulating the transcription levels of genes under stress conditions [32].

ROS significantly increased under abiotic stress, resulting in oxidative damage and leading to cell death [46]. Plants cope with environmental stress by developing various mechanisms to scavenge ROS to protect cells [47]. Proline and antioxidant enzymes are most important in ROS scavenging. Proline can prevent damage to cellular components caused by salt stress [48]. In this study, the proline content in the transgenic plants slightly increased under salt stress, suggesting that overexpression of *GhTRX134* might enhance salt tolerance via the proline pathway. This finding agrees with previous results that confirmed the positive role of proline in salt stress tolerance [49,50]. Plants have two defense mechanisms for scavenging ROS, including enzymatic and non-enzymatic mechanisms [51]. The antioxidant enzymes CAT, SOD, and POD act as the main ROS scavengers [47]. TRX genes regulate antioxidant enzymes [52]. In this study, our data indicated that the CAT, SOD, and POD activity levels were highly induced in the transgenic plants under drought stress. As previously mentioned, *CDSPs* regulate diverse pathways [53], and our results suggest that the overexpression of *GhTRX134* enhanced transgenic plant drought tolerance via enzymatic mechanisms and improved salt tolerance via the proline pathway.

## 5. Conclusions

In summary, we overexpressed *GhTRX134* in *Arabidopsis*, and the transgenic plants exhibited tolerance to abiotic stress. Moreover, silencing of *GhTRX134* caused cotton plants to become more sensitive to drought stress. The overexpression of *GhTRX134* enhanced stress-responsive genes under abiotic stress and the full-length *GhTRX134* gene acts as a transcriptional activator. Additionally, the transgenic plants exhibited increased enzyme activities and proline content under drought and salt stresses, respectively. Our findings suggest that *GhTRX134* is a key factor in abiotic stress responses. Therefore, the *GhTRX134* gene can be overexpressed in cotton plants to obtain transgenic lines for more functional details to detect the role of GhTRX134 in abiotic stress tolerance.

## Supplementary Materials

The following are available online at https://www.mdpi.com/2223-7747/9/10/1388/s1. Figure S1: The expression of *GhTRX134* in two-week-old transgenic and Wt plants under cold stress was monitored by qRT-PCR. The expression of *GhTRX134* in transgenic seedlings and Wt plants subjected to low temperature 4 °C for 72 h. Total RNA was isolated at the indicated times after treatments for qPCR analysis. Actin was used as a standard control in the experiments. The data represent the mean of three replicates. Error bars indicate ± SDs. Asterisks indicate significant differences (*** *p* < 0.001) compared to Wt as determined by Student’s *t*-test. Table S1: The GhTRX134 gene primers used for cloning, infusions, qPCR, VIGS, and Y2H. Table S2: Stress-responsive gene primers used for qRT-PCR. Table S3: In silico promoter analysis of *GhTRX134* (1500 bp region).

## Abbreviations

RPs: ribonucleoprotein, VIGS: virus-induced gene silencing, Wt: wild-type, CAT: catalase, MV: methyl viologen, ROS: reactive oxygen species, POD: peroxidase, SOD: superoxide dismutase, °C: degrees Celsius, aa: amino acid (s), At: *Arabidopsis thaliana* (*Arabidopsis*), bp: base pair, UTR: untranslated region, Col-0: Columbia wild-type, cm: centimeters, Kan: kanamycin, PEG: polyethylene glycol, qRT-PCR: quantitative real-time polymerase chain reaction, TF: transcription factor.
