# Supplementary material for: Overexpression of CDSP32 (GhTRX134) Cotton Gene Enhances Drought, Salt, and Oxidative Stress Tolerance in Arabidopsis
Source: Plants (Basel). 2020 Oct 19;9(10):1388. doi: 10.3390/plants9101388 (PMC7650641; doi:10.3390/plants9101388)
Supplement: Supplementary file 1 [file plants-09-01388-s001.zip › Supplemetry materials/Supplementary Table1.docx]

**Table S1***GhTRX134* gene’s primers used in cloning, infusions, qPCR, Virus induced gene silencing (VIGS), and Y2H

| **Code** | **Forward primer (5^’^→ 3^’^)** | **Reverse primer ( 5^’^→ 3^’^)** |
| --- | --- | --- |
| Cl-TRX134  q- TRX134  In-TRX134 | CAGTTTCTTTCTCACTTGTCTTCCC  GCGAGTTCAGAAGGTTCACAG  CA CGGGGGACTCTAGA CAGTTTCTTTCTCACTTGTCTTCCC | TGTATGAGTTACTTTTGAGCTTCCC  CGAGACTAGGGAGCTTTGCAA  GATCGGGGAAATTCGAGCTC TGTATGAGTTACTTTTGAGCTTCCC |
| VI-TRX134  Y-TRX134F  Y-TRX134C  Y-TRX134N | GGACTAGT GCGACTAGGCCCAGATCTTTT  CGGAATTCCAGTTTCTTTCTCACTTGTCTTCCC  CGGAATTCCAGCTGATGGGTGATGTGTTG  CGGAATTCTTCCGATCTTCACTTCACCCC | TTGGCGCGCC GAATGGCTGTCACCGTAGTAC  CGGGATCCTGTATGAGTTACTTTTGAGCTTCCC  CGGGATCCCCCAGATCCTACGTACCTTCC  CGGGATCCGGTCCGATCCCTTCTTCTTCA |

In: Infusions, Xba1: TCTAGA, Sac1: GAGCTC. VI: VIGS, Spe1: ACTAGT, Asc1: GGCGCGCC. Y: Y2H, EcoR1: GAATTC, BamH1: GGATCC. F: full gene. C: C- terminus. N: N-terminus
