# Supplementary material for: Overexpression of CDSP32 (GhTRX134) Cotton Gene Enhances Drought, Salt, and Oxidative Stress Tolerance in Arabidopsis
Source: Plants (Basel). 2020 Oct 19;9(10):1388. doi: 10.3390/plants9101388 (PMC7650641; doi:10.3390/plants9101388)
Supplement: Supplementary file 1 [file plants-09-01388-s001.zip › Supplemetry materials/Supplementary Table2.docx]

**Table S2** Responsive Gene’s primers used for qRT- PCR

| **Gene** | **Accession No** | **Forward primer (5^’^→ 3^’^)** | **Reverse primer ( 5^’^→ 3^’^)** |
| --- | --- | --- | --- |
| *AtRD22* | At5g25610 | GCGTTGGCAGCGGAAAA | GCGTTAGGATCGTCGTGG |
| *AtRD29A* | At5g52310 | CTGATCCCACCAAAGAAGAAACT | CTGATCCCACCAAAGAAGAAACT |
| *AtRAB18* | At5g66400 | AAGAAGAACATGGCGTCTTACC | GTTCCAAAGCCTTCAGTCCC |
| *AtERD15* | At2g41430 | CCAGCGAAATGGGGAAAC | ACAAAGGTACAGTGGTGGC |
| *AtKIN1* | At5g15960.1 | AAATGTCAGAGACCAACAAGAA | CTACTTGTTCAGGCCGGTCTT |
| Atactin | Atactin | GAAATCACAGCACTTGCACC | AAGCCTTTGATCTTGAGAGC |
