# Supplementary figures and images for: Overexpression of CDSP32 (GhTRX134) Cotton Gene Enhances Drought, Salt, and Oxidative Stress Tolerance in Arabidopsis
Source: Plants (Basel). 2020 Oct 19;9(10):1388. doi: 10.3390/plants9101388 (PMC7650641; doi:10.3390/plants9101388)

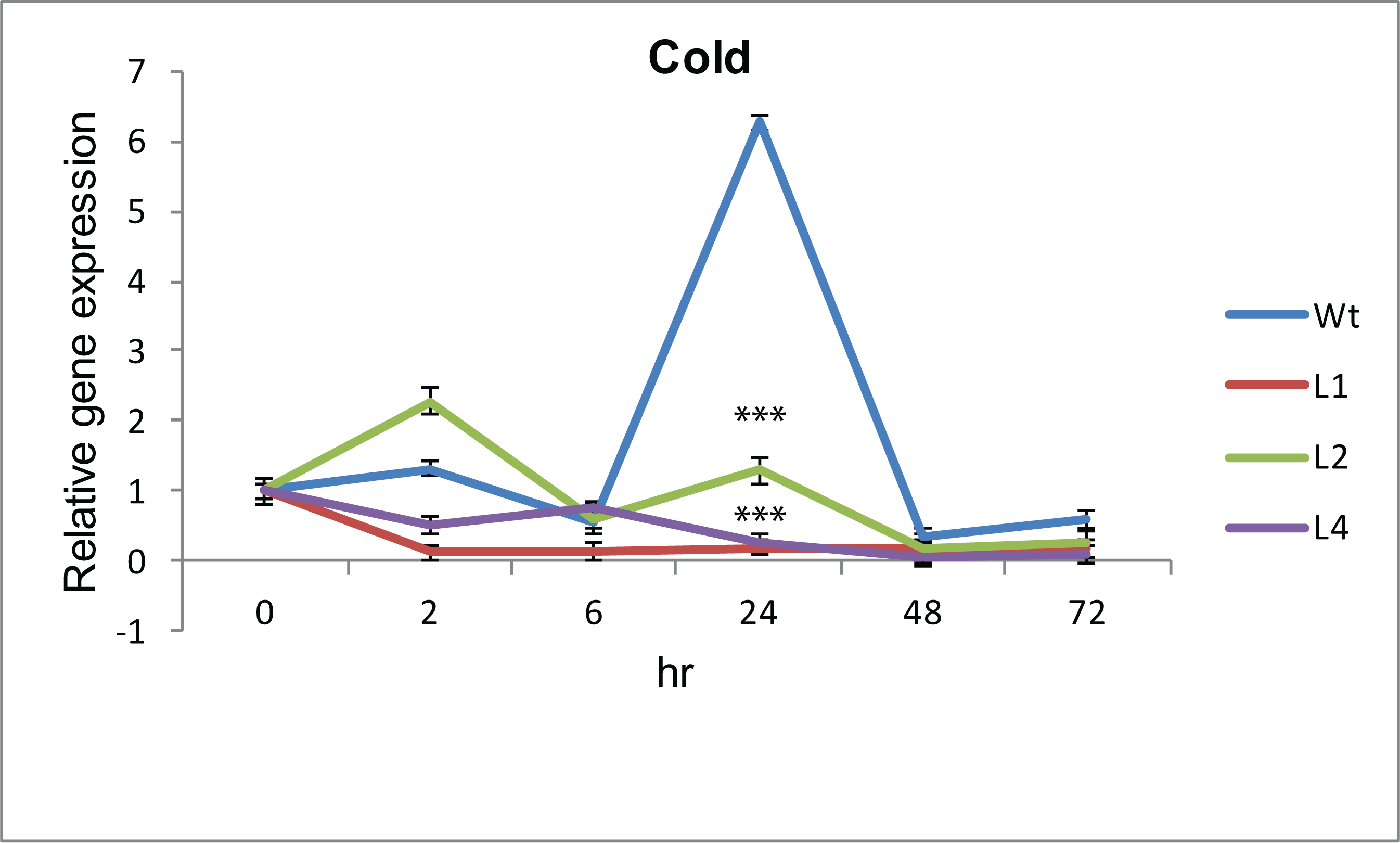

Supplement: Supplementary file 1 [file plants-09-01388-s001.zip › Supplemetry materials/Supplementary Figure 1.tif]
